# Functional *MAOB* Gene Intron 13 Polymorphism Predicts Dyskinesia in Parkinson's Disease

**DOI:** 10.1155/2022/5597503

**Published:** 2022-01-20

**Authors:** Matthias Löhle, Graziella Mangone, Wiebke Hermann, Denise Hausbrand, Martin Wolz, Julia Mende, Heinz Reichmann, Andreas Hermann, Jean-Christophe Corvol, Alexander Storch

**Affiliations:** ^1^Department of Neurology, University of Rostock, Rostock, Germany; ^2^German Centre for Neurodegenerative Diseases (DZNE) Rostock, Rostock, Germany; ^3^Sorbonne Université, INSERM UMRS1127 and CIC-1422, CNRS UMR7225, Assistance Publique Hôpitaux de Paris, ICM, Department of Neurology, Hôpital Pitié-Salpêtrière, Paris, France; ^4^Department of Neurology, Technische Universität Dresden, Dresden, Germany; ^5^Department of Neurology, Elblandklinikum Meißen, Meissen, Germany; ^6^Section for Translational Neurodegeneration “Albrecht Kossel”, Department of Neurology, University of Rostock, Rostock, Germany

## Abstract

Identification of individual risk factors for motor complications in Parkinson's disease (PD) can help to guide personalised medical treatment, particularly since treatment options are still limited. To determine whether common functional gene polymorphisms in the dopamine metabolism predict the onset of motor complications in PD, we performed a retrospective, observer-blinded follow-up study of 30 PD patients who underwent genotyping of dopa-decarboxylase (*DDC*; rs921451), monoamine oxidase B (*MAOB*; rs1799836), catechol-O-methyltransferase (*COMT*; rs4680), and dopamine transporter (*DAT*; variable number tandem repeat) polymorphisms. Onset of wearing-off and dyskinesias was determined by blinded clinical assessments. Predictive values of genotypes for motor complications were evaluated using Cox proportional hazard models. During a median follow-up time of 11.6 years, 23 (77%) of 30 PD patients developed wearing-off, 16 (53%) dyskinesias, and 23 (77%) any motor complication. The *MAOB* (rs1799836) polymorphism predicted development of dyskinesias with *MAOB*^*CC/(C)/CT*^ genotypes (resulting in low/intermediate brain enzyme activity) being associated with lower hazard ratios (unadjusted HR [95% CI]: 0.264 [0.089–0.787]; *p*=0.012; adjusted HR [95% CI]: 0.142 [0.039–0.520]; *p*=0.003) than *MAOB*^*TT/(T)*^ genotypes (resulting in high brain enzyme activity). *DDC* (rs921451), *COMT* (rs4680), and *DAT* (VNTR) polymorphisms were not predictive of motor complications. Together, the *MAOB* (rs1799836) polymorphism predicts the development of dyskinesias in PD patients. Our results need confirmation in larger cohorts. If confirmed, individual assessment of this polymorphism might be helpful for early risk stratification and could comprise a step towards patient-tailored therapeutic strategies to prevent or delay motor complications in the course of PD.

## 1. Introduction

Parkinson's disease (PD) is associated with motor complications, such as wearing-off and levodopa-induced dyskinesia (LID), which affect about 50% of patients after 5 years of treatment. Their pathophysiology involves presynaptic mechanisms [1] and postsynaptic factors downstream to the nigrostriatal dopaminergic input into the striatum [2]. Moreover, it has been speculated that early compensatory changes in dopamine metabolism, such as an increase in dopamine turnover, could predispose for motor complications in PD [3].

Several cross-sectional studies have tried to elucidate whether genetic variants may influence the susceptibility for LID, but have often yielded inconsistent results possibly due to differences in screening methods and to comparison of data derived from a large variety of genetically and ethnically diverse populations [4]. For example, some investigations into genetic variants of the dopamine receptor D2 (DRD2) gene reported an association with dyskinesia [5–8], whereas others did not identify an association between DRD2 genotypes and the risk for dyskinesia [9–14]. Consequently, a recent systematic review and meta-analysis of the existing studies concluded that the role of genetic factors for individual susceptibility to LID remains unclear and that further studies are required [4].

Using quantitative ^18^F-fluorodopa PET imaging, we have recently shown that the monoamine oxidase B gene intron 13 polymorphism (*MAOB*; rs1799836) predicts dopamine turnover in *de novo* PD with the *MAOB*^*CC/(C)/CT*^ genotypes leading to lower dopamine turnover [15], which in turn has been associated with a decreased risk for motor complications [16]. Conversely, functional polymorphisms in dopa-decarboxylase (*DDC*; rs921451), catechol-O*-*methyl transferase (*COMT*; rs4680), and dopamine transporter (*DAT*; variable number tandem repeats) were not predictive of dopamine turnover in our previous PET study [15]. Thus, we now aimed to analyse in this well-defined, longitudinally followed cohort [15–17] whether common functional polymorphisms of *MAOB* (rs1799836), *COMT* (rs4680), *DDC* (rs921451), and *DAT* (VNTR) were predictive of motor complications. We hypothesized that *MAOB*^*CC/(C)/CT*^ genotypes in the intron 13 polymorphism, which encode lower enzyme activity [18] and hence lower dopamine turnover [15], would be associated with a lower risk for motor complications.

## 2. Materials and Methods

**Figure 1:**
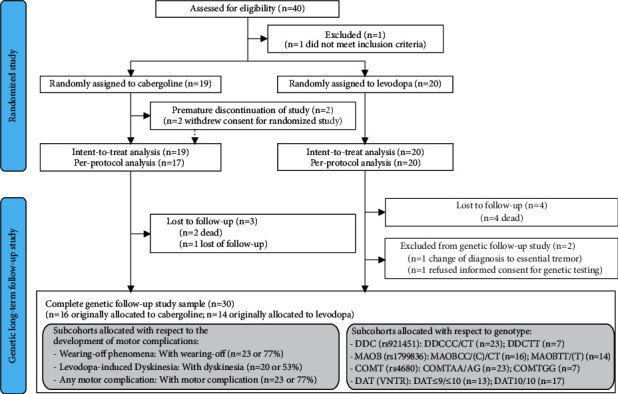
CONSORT flow diagram for the study.

This is a retrospective analysis of data from a longitudinal study, which included participants with PD from a previous clinical trial [17], who were meticulously followed up over 11 years by movement disorder specialists and underwent genotyping of common polymorphisms of genes involved in dopamine metabolism (Figure 1). Major eligibility criteria of the initial study [17] were age between 40 and 85 years, diagnosis of PD according to UK Brain Bank criteria [19], and a rating on the modified Hoehn and Yahr scale of 1–2.5 [20]. Patients were excluded if they had been exposed to any dopaminergic therapy prior to study inclusion. Follow-up evaluations in our outpatient clinic were performed every three to six months depending on the clinical needs of individual patients.

Motor symptoms and activities of daily living were estimated using the Unified PD Rating Scale (UPDRS), which was assessed by movement disorder trained physicians blinded to the genotyping data. Onset of wearing-off was classified as the first time patients or medical records indicated a predictable recurrence of motor symptoms preceding scheduled doses of antiparkinsonian medication and usually improving after those doses. Onset of LID was defined as the first time patients or treating neurologists noted involuntary movements of face, trunk, or extremities following levodopa intake. Genotyping of the common functional polymorphisms *DDC* (rs921451), *MAOB* (rs1799836), *COMT* (rs4680), and *DAT* (VNTR) was performed as described previously [15]. The study was approved by the Ethics Committee at TU Dresden (EK91052003) and originally registered with ClinicalTrials.gov (NCT00153972).

Statistical analyses were performed with SPSS software, version 23.0 (SPSS, Chicago, IL). Comparisons of clinical data were made with the unpaired *t*-test or Mann–Whitney *U*-test or Fisher exact tests, as appropriate. To examine the risks for motor complications, we used univariate and multivariate Cox proportional hazards models to estimate hazard ratios (HRs) with 95% confidence intervals (95% CIs) and *p* values for pairwise comparisons. The proportional hazards assumption was tested using log-log plots. Similar to previous studies [16, 21–23], gender, age at symptom onset, disease duration at baseline, baseline UPDRS ADL and motor scores, baseline weight, treatment allocation (initial randomization to cabergoline/levodopa), and antiparkinsonian therapy at time of motor complication onset (antiparkinsonian agents) were entered into the multivariate Cox proportional hazards model to adjust for relevant covariates. A stepwise selection process, with significance levels for both entry and retention in the model set at 0.05, was used to select the most significant predictors. In order to assess the risk for motor complications, patients were dichotomized into low/intermediate enzyme/transporter activity and high enzyme/transporter activity as displayed in Supplementary Table S1. Genotypes with high enzyme/transporter activity were designated as reference groups (relative risk = 1). Significance level (two-tailed) was set at *p* < 0.0125 to correct for multiple comparisons using the Bonferroni method.

## 3. Results

Sufficient datasets were available from 30 patients (20 (67%) males and 10 (33%) females; mean age at PD onset: 59.8 ± 9.1 years; age at baseline: 61.0 ± 9.5 years; disease duration at baseline: 0.3 ± 0.7 years [range: 0.0–3.0]; baseline UPDRS III motor score: 19.3 ± 7.4). Median (interquartile range) follow-up period was 11.6 (11.0–12.3) years. For details on demographic and clinical characteristics of the cohort, refer to Supplementary Table S2. Allele frequencies for all polymorphisms were within the range of frequencies reported in databases, and all genotypes were in the Hardy–Weinberg equilibrium (Supplementary Table S1). At last follow-up, 23 patients (77%) had developed wearing-off, 16 (53%) LID, and 23 (77%) any motor complication. We found no significant differences in demographic, clinical characteristics, initial response to therapy [17], levodopa equivalent daily doses, and PD medication classes between genotype groups (Supplementary Table S2).

Univariate Cox proportional hazard models demonstrated that patients with *MAOB*^*CC/(C)/CT*^ genotypes (low/intermediate enzyme activity) had a lower risk for LID, but not for wearing-off or any motor complication, compared to individuals with *MAOB*^*TT/(T)*^ genotypes (high enzyme activity) (Table 1). *DDC*, *COMT*, and *DAT* polymorphisms were not predictive of motor complications. Multivariate Cox proportional hazard models adjusting for relevant covariates confirmed these results (Table 1).

**Figure 2:**
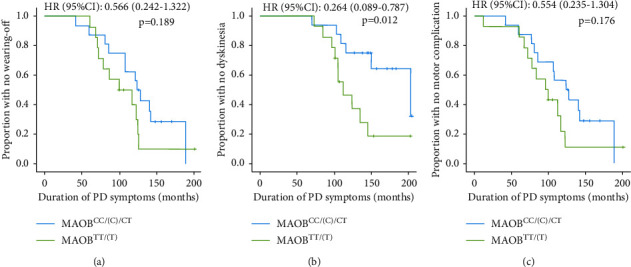
Risk of levodopa-induced motor complications with respect to genotypes in the *MAOB* rs1799836 polymorphism. Kaplan–Meier curves show the association between *MAOB* rs1799836 genotypes (*MAOB*^*CC/(C)/CT*^ genotypes: associated with low/intermediate enzyme activity in the brain (blue line); *MAOB*^*TT/(T)*^ genotypes: associated with high enzyme activity in the brain (green line)) and the risk for wearing-off (a), levodopa-induced dyskinesia (b), and any motor complication (c). Hazard ratios (HR) with 95% confidence intervals (95% CI) and *p* values are from univariate Cox proportional hazard models with those genotypes associated with high MAOB activity (*MAOB*^*TT/(T)*^) adopted as the reference group.

Kaplan–Meier curves revealed that patients with *MAOB*^*CC/(C)/CT*^ genotypes (intermediate/low enzyme activity) had a higher chance to remain free from LID, whereas individuals with *MAOB*^*TT/(T)*^ genotypes (high enzyme activity) were more likely to experience LID earlier (Figure 2). These effects were again not observed for the occurrence of wearing-off and any motor complication (Figures 2(a) and 2(c)).

## 4. Discussion

This is the first longitudinal study to demonstrate that the *MAOB* rs1799836 polymorphism predicts the development of LID in PD, with a lower risk observed in patients carrying the *MAOB*^*CC/(C)/CT*^ genotype (associated with low/intermediate brain enzyme activity). In contrast, other functional gene polymorphisms involved in the dopamine metabolism were not predictive of motor complications.

By applying a longitudinal study design in meticulously followed and evenly matched patients, we found that *MAOB*^*TT/(T)*^ genotypes in rs1799836 polymorphism encoding for higher MAOB activity predispose for earlier LID. Our results are in agreement with a cross-sectional, retrospective study from Brazil that reported a higher risk for LID with the *MAOB*^*AA/(A)*^ genotype (corresponding to *MAOB*^*TT/(T)*^ in our study), but was restricted to levodopa-treated patients [24]. Another cross-sectional study from China found a higher frequency of the *MAOB*^*AG*^ genotype (corresponding to *MAOB*^*CT*^ in our study) in dyskinetic patients, but failed to identify differences in allele frequencies [25]. However, dyskinetic patients in that study had a younger age of onset, longer disease duration, and higher UPDRS part III scores, which are known risk factors for dyskinesia and could only be adjusted for statistically. Using retrospective clinical data from medical records of 110 Asian patients with PD, a recent Japanese study conversely to our findings suggested that PD patients carrying the G allele (AG or GG, corresponding to *MAOB*^*CC/(C)/CT*^ in our study) exhibit an approximately 3-fold increase in the occurrence of dyskinesia [26]. However, there was a nonsignificant trend (*p*=0.069) for higher total LED in patients with AG and GG genotypes in this study, which may have contributed to the higher dyskinesia risk observed in these genotype groups. Conflicting results have also been reported for rs4680 in the *COMT* gene [24, 25, 27–32] and variable number tandem repeats in the *DAT* gene [11, 12, 33], whereas the predictive value of the rs921451 polymorphism in the *DDC* gene for motor complications had not been examined prior to our study. Recently, a systematic review and meta-analysis suggested that the conflicting results of genetic studies may have been caused by differences in the screening methods and in the comparison of the clinical data in genetically and ethnically diverse populations and eventually concluded that the role of genetic factors in susceptibility to dyskinesia remains unclear [4].

Biochemical data and previous PET data from our cohort help to elucidate potential mechanisms, which could mediate the influence of the *MAOB* rs1799836 genotype on the time to onset of LID. *MAOB*^*CC/(C)/CT*^ genotypes correlate with low/intermediate enzyme activity in human brain tissue [18] and with low putaminal dopamine turnover in early PD as directly measured by ^18^F-fluorodopa PET [15]. Extensively elevated dopamine turnover has been discussed as an early disease-intrinsic compensatory mechanism leading to imbalance between dopamine synthesis, storage, and release, subsequently to more prominent fluctuations in dopamine concentration and thus greater propensity for motor complications [3]. In line with our previous results showing no influence of polymorphisms in *COMT* (rs4680), *DAT* (VNTR), and *DDC* (rs921451) on dopamine turnover in *de novo* PD [15], we did not observe any associations between these polymorphisms and motor complications. It is thus likely that the effects of the *MAOB* rs1799836 genotype on the onset of levodopa-induced motor complications are mediated by a modulation of compensatory changes in dopamine metabolism.

Our study has several limitations. First, the use of a relatively small cohort from a single centre and the retrospective nature of our study may limit generalizability. On the contrary, our results were obtained from a meticulously followed up and well-defined patient cohort with available ^18^F-fluorodopa PET data, exclusively allowing for mechanistic considerations of genotype effects. Secondly, we were not able to control for different medication regimes during the observation period, since the treatment was open-label after the initial allocation. However, we did not find any differences in the pharmacological treatment between the genotype groups, which argues against a differential effect of medication on study outcomes.

## 5. Conclusion

In conclusion, our study suggests that the functional *MAOB* gene intron 13 polymorphism could predict the development of LID. However, it is essential that these results are confirmed by other studies on larger cohorts, especially as our study cohort is relatively small and the results of previous genetic studies have been conflicting. Individual assessment of this polymorphism might be helpful for early risk stratification and could aid patient-tailored therapeutic strategies to prevent or delay motor complications in the course of PD.

## Figures and Tables

**Table 1:** Cox proportional hazard ratios estimating the risk for motor complications with respect to genotypes in functional polymorphisms associated with lower enzyme/transporter activity^a^.

	Wearing-off				Levodopa-induced dyskinesia				Any motor complication	
	HR (95% CI)^b^	*p*	Adjusted HR (95% CI)^b,c^	*p*	HR (95% CI)^b^	*p*	Adjusted HR (95% CI)^b,c^	*p*	HR (95% CI)^b,c^	*p*
*DDC* ^*CC/CT*^ (rs921451)	0.569 (0.218–1.488)	0.250	0.633 (0.240–1.668)	0.355	0.699 (0.218–2.244)	0.548	0.801 (0.237–2.705)	0.721	0.514 (0.196–1.350)	0.177
*MAOB* ^*CC/(C)/CT*^ (rs1799836)	0.566 (0.242–1.322)	0.189	0.197 (0.563–1.347)	0.197	0.264 (0.089–0.787)	**0.012**	0.142 (0.039–0.520)	**0.003**	0.554 (0.235–1.304)	0.176
*COMT* ^*AA/AG*^ (rs4680)	1.216 (0.410–3.607)	0.725	1.277 (0.430–3.789)	0.660	5.526 (0.725–42.095)	0.099	5.298 (0.677–41.484)	0.112	1.280 (0.432–3.795)	0.656
*DAT* ^*≤9/≤10*^ (VNTR)	0.869 (0.370–2.039)	0.746	1.081 (0.452–2.582)	0.861	0.943 (0.335–2.655)	0.912	1.108 (0.372–3.295)	0.854	0.994 (0.422–2.338)	0.988

HR: hazard ratio; 95% CI: 95% confidence interval. ^a^Patients were grouped into low/intermediate enzyme/transporter activity (*DDC*^*CC/CT*^ (*n* = 23); *MAOB*^*CC/(C)/CT*^ (*n* = 16); *COMT*^*AA/AG*^ (*n* = 23); *DAT*^*≤9/≤10*^ (*n* = 13)) and high enzyme/transporter activity (*DDC*^*TT*^ (*n* = 7); *MAOB*^*TT/(T)*^ (*n* = 14); C*OMT*^*GG*^ (*n* = 7); *DAT*^*10/10*^ (*n* = 17)). Refer to Supplementary Table S1 for details. ^b^An HR < 1 indicates smaller risk for motor complications with genotypes resulting in low/intermediate enzyme/transporter activity (*DDC*^*CC/CT*^ rs921451 genotype; *MAOB*^*CC/(C)/CT*^ rs1799836 genotype; *COMT*^*AA/AG*^ rs4680 genotype; *DAT*^*≤9/≤10*^ V NTR genotype). ^c^Adjustment of HR for relevant covariates was performed by using multivariate Cox proportional hazard models using a stepwise selection process retaining as final variables disease duration at baseline for the prediction of wearing-off, gender, disease duration at baseline, and MAOB-I therapy for levodopa-induced dyskinesia and no variables for any motor complication (no adjustment needed).

## Data Availability

The data used to support the findings of this study are available from the corresponding authors upon request.

## Supplementary Materials

Supplementary Materials Supplementary Table S1: characteristics of selected genetic variants. Supplementary Table S2: demographic and clinical characteristics of the study participants with respect to genetic polymorphisms.
