# Supplementary material for: Functional MAOB Gene Intron 13 Polymorphism Predicts Dyskinesia in Parkinson's Disease
Source: Parkinsons Dis. 2022 Jan 20;2022:5597503. doi: 10.1155/2022/5597503 (PMC8794697; doi:10.1155/2022/5597503)
Supplement: Supplementary Materials — Supplementary Table S1: characteristics of selected genetic variants. Supplementary Table S2: demographic and clinical characteristics of the study participants with respect to genetic polymorphisms. [file 5597503.f1.docx]

**Supplementary Materials**

**Functional *MAOB* gene intron 13 polymorphism predicts dyskinesia in Parkinson’s disease**

Matthias Löhle, Graziella Mangone, Wiebke Hermann, Denise Hausbrand, Martin Wolz, Julia Mende, Heinz Reichmann, Andreas Hermann, Jean-Christophe Corvol, and Alexander Storch

**Supplementary Tables:**

**Supplementary Table S1.** Characteristics of selected genetic variants.

**Supplementary Table S2.** Demographic and clinical characteristics of study participants with respect to genetic polymorphisms.

**Supplementary Table S1. Characteristics of selected genetic variants**

| **Gene** | **Variant** | **Alleles** | **Location in gene** | **MAF^a^** | **Allele frequency in cohort** | **Functional consequence** |
| --- | --- | --- | --- | --- | --- | --- |
| *DDC* | rs921451 | C/T | Intron | C=0.346 | C=0.517 | C=low response to levodopa suggesting low enzyme activity^1^ |
| *MAOB* | rs1799836 | C/T | Intron | C=0.457 | C=0.467 | C=low enzyme activity in the brain^2^ |
| *COMT* | rs4680 | A/G | Exon, missense | A=0.369 | A=0.450 | A=low enzyme activity^3^ |
| *DAT (SLC6A3)* | VNTR | 3-, 7-, 8-, 9-, 10-, 11-repeats | UTR’3 | 10: 0.654-0.900 9: 0.100-0.330 7: 0.000-0.010 | 10 repeats: 0.783 9 repeats: 0.200 7 repeats: 0.017 | 10 repeats=high expression of DAT protein leading to high transporter activity^4-6^ |

*COMT*: Gene encoding catechol-O-methyltransferase. *DAT:* Gene encoding dopamine transporter (DAT). *DDC*: Gene encoding dopa-decarboxylase; MAF: minor allele frequency as reported in DBSNP database. *MAOB:* Gene encoding monoamine oxidase B (MAO-B). UTR’3: three prime untranslated region. VNRT: Variable number tandem repeat. Table adapted from Löhle M, *et al.* Mov Disord. 2018;33(9):1496-1501.

^a^In case of the DAT variant, the allele frequencies of the most frequent alleles in European populations are presented as reported in the ALFRED database of the US National Science Foundation.

References: ^1^Devos *et al.* Parkinsonism Relat Disord 2014;20:170-175. ^2^Balciuniene *et al.* Hum Genet 2002;110:1-7. ^3^Syvanen *at al.* Pharmacogenetics 1997;7:65-71. ^4^Heinz *et al.* Neuropsychopharmacology 2000;22:133-139. ^5^Mill *et al.* Am J Med Genet 2002;114:975-979. ^6^VanNess *et al.* BMC Genet 2005;6:55.

**Supplementary Table S2. Demographic and clinical characteristics of study participants with respect to genetic polymorphisms**

|  | **Overall cohort** | ***DDC^CC/CT^*** | | ***DDC^TT^*** | | | ***p*** | | ***MAOB^CC/(C)/CT^*** | | ***MAOB^TT/(T)^*** | ***p*** | ***COMT^AA/AG^*** | ***COMT^GG^*** | ***p*** | ***DAT^≤9/≤10^*** | ***DAT^10/10^*** | ***p*** | |
| --- | --- | --- | --- | --- | --- | --- | --- | --- | --- | --- | --- | --- | --- | --- | --- | --- | --- | --- | --- |
| ***Participants (n)*** | 30 | 23 | | 7 | | |  | | 16 | | 14 |  | 23 | 7 |  | 13 | 17 |  | |
| ***Demographics at clinical data at baseline*** | | |  | |  |  | |  | |  |  |  |  |  |  |  |  |  |  |
| Men/women | 20 (67%)/ 10 (33%) | 16 (70%)/ 7 (30%) | | 4 (57%)/ 3 (43%) | | | 0.657^#^ | | 10 (60%)/ 6 (40%) | | 10 (71%)/ 4 (29%) | 0.709^#^ | 17 (74%)/ 6 (26%) | 4 (57%)/ 3 (43%) | 0.181^#^ | 9 (69%)/ 4 (31%) | 11 (65%)/ 6 (35%) | 1.000^#^ | |
| Age (years) | 61.0±9.5 | 61.5±9.6 | | 59.6±9.5 | | | 0.665^§^ | | 61.6±11.0 | | 60.4±7.8 | 0.731^§^ | 60.5±8.3 | 62.6±13.3 | 0.618^§^ | 62.7±9.3 | 59.7±9.7 | 0.394^§^ | |
| Age at symptom onset (years) | 58.2±9.6 | 58.3±9.8 | | 58.0±9.5 | | | 0.943^§^ | | 58.1±11.2 | | 58.4±7.8 | 0.949^§^ | 57.8±8.7 | 59.7±12.8 | 0.649^§^ | 59.9±11.1 | 56.9±8.4 | 0.408^§^ | |
| Age at diagnosis (years) | 59.8±9.1 | 60.1±9.0 | | 58.7±9.7 | | | 0.724^§^ | | 60.0±10.2 | | 59.6±8.0 | 0.900^§^ | 59.5±8.1 | 60.9±12.4 | 0.731^§^ | 62.0±9.7 | 58.1±8.5 | 0.251^§^ | |
| Duration of PD at baseline (years)^&^ | 0.3±0.7 | 0.1±0.5 | | 0.6±1.1 | | | 0.135^§^ | | 0.2±0.5 | | 0.3±0.8 | 0.700^§^ | 0.3±0.8 | 0.0±0.0 | 0.069^§^ | 0.2±0.6 | 0.2±0.8 | 0.986^§^ | |
| Symptom duration at baseline (years) | 1.8±2.0 | 1.8±2.0 | | 1.3±1.7 | | | 0.530^§^ | | 1.9±2.2 | | 1.5±1.7 | 0.485^§^ | 1.9±2.1 | 1.1±1.1 | 0.397^§^ | 2.2±2.6 | 1.3±1.3 | 0.242^§^ | |
| Baseline weight, kg | 76.4±13.6 | 76.8±14.5 | | 75.0±11.3 | | | 0.769^§^ | | 78.2±16.5 | | 74.3±9.7 | 0.449^§^ | 75.3±10.6 | 79.9±21.7 | 0.443^§^ | 78.3±14.4 | 74.9±13.3 | 0.525^§^ | |
| Modified Hoehn & Yahr stage at baseline | 2.0 (1.0-2.0) | 2.0 (1.0-2.0) | | 2.0 (1.0-2.0) | | | 0.826^#^ | | 2.0 (1.0-2.0) | | 2.0 (1.5-2.0) | 0.521^#^ | 2.0 (1.0-2.0) | 2.0 (1.0-2.1) | 0.826^#^ | 2.0 (1.0-2.0) | 2.0 (1.0-2.0) | 1.000^#^ | |
| Baseline UPDRS part II ADL score | 6.7±3.0 | 7.0±3.2 | | 4.7±1.1 | | | 0.089^§^ | | 5.6±2.0 | | 7.5±3.6 | 0.101^§^ | 6.9±3.2 | 5.1±1.6 | 0.061^§^ | 6.1±2.8 | 6.8±3.1 | 0.508^§^ | |
| Baseline UPDRS part III motor score | 19.3±7.4 | 19.7±8.2 | | 17.9±4.3 | | | 0.575^§^ | | 17.1±6.6 | | 21.7±7.8 | 0.092^§^ | 20.0±7.9 | 16.7±5.2 | 0.307^§^ | 19.1±5.9 | 19.4±8.6 | 0.905^§^ | |
| Initial treatment allocation, levodopa/cabergoline | 14 (47%)/ 16 (53%) | 11 (48%)/ 12 (52%) | | 3 (43%)/ 4 (57%) | | | 1.000^#^ | | 8 (55%)/ 7 (47%) | | 6 (46%)/ 7 (54%) | 1.000^#^ | 12 (52%)/ 11 (48%) | 2 (29%)/ 5 (61%) | 0.399^#^ | 5 (38%)/ 8 (62%) | 9 (53%)/ 8 (47%) | 0.484^#^ | |

Data are mean ± standard deviations, median (interquartile range) or numbers (%). ADL: Activities of daily living. LED: Levodopa equivalent dose calculated according to Tomlinson *at al.* Mov Disord 2010;25:2649-2653. COMT: Catechol-O-Methyltransferase. DAT: Dopamine transporter. DDC: Dopa decarboxylase. MAOB: Monoamine oxidase B. PD: Parkinson’s disease. UPDRS: Unified Parkinson’s disease rating scale. *p* values are from ^#^Fisher’s exact test, ^$^Mann-Whitney U test, or ^§^unpaired two-sided t-test comparing selected enzyme/transporter alleles. ^&^Disease duration was defined as the time since PD had first been diagnosed.

**Supplementary Table S2 (cont.). Demographic and clinical characteristics of study participants with respect to genetic polymorphisms**

|  | **Overall cohort** | ***DDC^CC/CT^*** | ***DDC^TT^*** | | | ***p*** | | ***MAOB^CC/(C)/CT^*** | ***MAOB^TT/(T)^*** | | ***p*** | | ***COMT^AA/AG^*** | | | ***COMT^GG^*** | | ***p*** | | ***DAT^≤9/≤10^*** | | ***DAT^10/10^*** | ***p*** |
| --- | --- | --- | --- | --- | --- | --- | --- | --- | --- | --- | --- | --- | --- | --- | --- | --- | --- | --- | --- | --- | --- | --- | --- |
| ***Clinical data at last follow-up*** | |  | |  |  | |  |  |  | |  | |  | | |  | |  | |  | |  |  |
| Follow-up period, years | 11.6 (11.0-12.3) | 11.7 (11.2-12.7) | 11.1 (7.2-11.6) | | | 0.174^$^ | | 11.4 (10.9-12.2) | 11.6 (10.2-12.4) | | 0.790^$^ | | 11.6 (11.1-12.7) | | | 11.4 (8.4-11.7) | | 0.820^$^ | | 11.5 (8.0-12.0) | | 11.7 (11.2-12.7) | 0.363^$^ |
| Modified Hoehn & Yahr stage at baseline | 3.0 (2.5-4.0) | 3.0 (2.5-4.0) | 3.0 (2.0-4.0) | | | 1.000^#^ | | 3.0 (2.5-4.0) | 3.0 (2.3-4.0) | | 1.000^#^ | | 3.0 (2.5-4.0) | | | 4.0 (2.0-5.0) | | 0.140^#^ | | 4.0 (2.5-4.5) | | 3.0 (2.5-3.0) | 0.023^#^ |
| Follow-up UPDRS part II ADL score (n=21) | 17.1±5.6 | 16.9±6.3 | 17.3±4.0 | | | 0.887^§^ | | 16.8±7.4 | 17.3±2.9 | | 0.850^§^ | | 16.5±4.9 | | | 18.8±7.9 | | 0.438^§^ | | 18.7±5.3 | | 14.9±5.8 | 0.130^§^ |
| Follow-up UPDRS part III motor score (n=19) | 33.6±12.7 | 32.9±13.9 | 35.0±10.5 | | | 0.750^§^ | | 33.2±13.8 | 33.9±12.3 | | 0.911^§^ | | 32.4±11.3 | | | 36.8±16.9 | | 0.523^§^ | | 35.9±14.2 | | 30.4±10.1 | 0.361^§^ |
| Total LED per day (mg) | 876±350 | 877±338 | 873±412 | | | 0.959^$^ | | 850±354 | 902±357 | | 0.635^$^ | | 930±363 | | | 678±220 | | 0.157^$^ | | 859±440 | | 888±279 | 0.568^$^ |
| ***Antiparkinsonian medication at time of first motor complication or last of follow-up*** | | | | | | | | | |  | |  |  |  |  |  |  |  |  |  |  |  |  |
| Levodopa therapy, n | 25 (83%) | 18 (78%) | 7 (100%) | | | 0.304^#^ | | 12 (75%) | 13 (93%) | | 0.336^#^ | | 20 (87%) | | | 5 (71%) | | 0.565^#^ | | 10 (77%) | | 15 (88%) | 0.628^#^ |
| Dopamine agonist, n | 26 (87%) | 20 (87%) | 6 (86%) | | | 1.000^#^ | | 14 (88%) | 12 (86%) | | 1.000^#^ | | 21 (91%) | | | 5 (71%) | | 0.225^#^ | | 12 (92%) | | 14 (82%) | 0.613^#^ |
| MAO-B inhibitor, n | 14 (47%) | 11 (48%) | 3 (43%) | | | 1.000^#^ | | 10 (63%) | 4 (29%) | | 0.081^#^ | | 12 (52%) | | | 2 (29%) | | 0.399^#^ | | 8 (62%) | | 6 (35%) | 0.269^#^ |
| COMT inhibitor, n | 4 (13%) | 2 (9%) | 2 (29%) | | | 0.225^#^ | | 1 (6%) | 3 (21%) | | 0.315^#^ | | 4 (17%) | | | 0 (0%) | | 0.548^#^ | | 0 (0%) | | 4 (24%) | 0.113^#^ |
| Amantadine, n | 9 (30%) | 7 (30%) | 2 (29%) | | | 1.000^#^ | | 4 (36%) | 5 (46%) | | 0.649^#^ | | 7 (30%) | | | 2 (29%) | | 1.000^#^ | | 4 (31%) | | 5 (29%) | 1.000^#^ |

Data are mean ± standard deviations, median (interquartile range) or numbers (%). ADL: Activities of daily living. LED: Levodopa equivalent dose calculated according to Tomlinson *at al.* Mov Disord 2010;25:2649-2653. COMT: Catechol-O-Methyltransferase. DAT: Dopamine transporter. DDC: Dopa decarboxylase. MAOB: Monoamine oxidase B. PD: Parkinson’s disease. UPDRS: Unified Parkinson’s disease rating scale. *p* values are from ^#^Fisher’s exact test, ^$^Mann-Whitney U test, or ^§^unpaired two-sided t-test comparing selected enzyme/transporter alleles. ^&^Disease duration was defined as the time since PD had first been diagnosed.
